# Improved downstream functional analysis of single-cell RNA-sequence data using DGAN

**DOI:** 10.1038/s41598-023-28952-y

**Published:** 2023-01-28

**Authors:** Diksha Pandey, Perumal P. Onkara

**Affiliations:** Department of Biotechnology, National Institute of Technology, Warangal, India

**Keywords:** Computational models, Data acquisition, Data processing, Machine learning, Programming language, Sequence annotation, Software, Statistical methods

## Abstract

The dramatic increase in the number of single-cell RNA-sequence (scRNA-seq) investigations is indeed an endorsement of the new-fangled proficiencies of next generation sequencing technologies that facilitate the accurate measurement of tens of thousands of RNA expression levels at the cellular resolution. Nevertheless, missing values of RNA amplification persist and remain as a significant computational challenge, as these data omission induce further noise in their respective cellular data and ultimately impede downstream functional analysis of scRNA-seq data. Consequently, it turns imperative to develop robust and efficient scRNA-seq data imputation methods for improved downstream functional analysis outcomes. To overcome this adversity, we have designed an imputation framework namely deep generative autoencoder network [DGAN]. In essence, DGAN is an evolved variational autoencoder designed to robustly impute data dropouts in scRNA-seq data manifested as a sparse gene expression matrix. DGAN principally reckons count distribution, besides data sparsity utilizing a gaussian model whereby, cell dependencies are capitalized to detect and exclude outlier cells via imputation. When tested on five publicly available scRNA-seq data, DGAN outperformed every single baseline method paralleled, with respect to downstream functional analysis including cell data visualization, clustering, classification and differential expression analysis. DGAN is executed in Python and is accessible at https://github.com/dikshap11/DGAN.

## Introduction

More recently next-generation sequencing (NGS) technologies are increasingly being adopted as a versatile and expedient tool for an assortment of functional genomics applications including RNA-sequencing and single-cell RNA-sequence (scRNA-seq)^1^. While NGS technologies continue to endure transformation of becoming a mainstream investigational tool at the same time the volume of scRNA-seq data has also risen dramatically over the last few years^2^. Despite the fact that the initial pioneering investigation of scRNA sequencing was published more than a decade ago^3^subsequent studies over the course of the decade have ameliorated several characteristics of capturing RNA expression at the single-cell level. Besides acquiring transcriptome-wide expression counts of tens of hundreds of individual cells, variability with high resolution of cellular differences, investigations also have decrypted the dynamics of heterogeneous cell classifications, complex tissues within the microenvironment^4,5^. On the whole, purpose of scRNA-seq data analysis is to detect stimulating cell conditions that prevail in the biological samples, while cells are clustered according to cell to cell similarity within gene expression profiles^6^.

Nevertheless, the increasing number of biological cells, high dropout rates and technical noise levels create considerable computational challenges in downstream functional analysis of scRNA-seq data^7^. In addition, these challenges also compromise the competence to extract the plenty of information available besides suffering from execution time, accuracy and scalability issues. As both data volume and data complexity of scRNA-seq are expanding exponentially, thus more robust imputation approaches become indispensable for downstream functional analysis.

While technological improvements in high-throughput scRNA-seq technologies have facilitated the quantity of gene expressions profiles individual cells, thereby unfolding new insights at the genomic scale that were previously concealed in gene expression analysis executed by bulk RNA sequencing^8,9^ conversely, scRNA-seq data quality is more often much less than that of bulk RNA sequencing data^10^ as the former data is particularly noisy due to technical besides biological error. High noise levels in scRNA-seq data are largely attributed to inadequate RNA input in addition to low quantities of RNA that are frequently observed during the reverse transcription phase in scRNA-seq investigations, denoted as ‘dropouts’. Dropouts are either categorized as technical /true zero counts or as false negatives depending on whether or not they arise due to amplification failure of original RNA transcripts during the sequencing step^11^. Technical/true zero counts (also called as missing values) arise due to genes that aren't expressed, as opposed to false zero counts, that are caused by measurement errors. Missing values are more likely to occur if gene expression is significantly high in some, but not in other identical type of cells. Likewise, missing values are quite frequent in scRNA-seq data attained from lower-level gene expression of RNA transcripts with relatively shallow sequencing depths^12^. Further, incidence of missing values often hinders particularly downstream functional analysis of scRNA-seq data^13^, including cell data visualization, cell clustering, classification and differential expression analysis^14^.

In the recent past, a multitude of imputation models have been implemented. Data imputation models amongst several others such as ScImpute^15^, SAVER^16^, MAGIC^17^, AutoImpute^18^, VIPER^19^, DrImpute^20^ and scMTD^21^ acquire their inputs from the entire gene set thereby attaining accurate and denoised expression estimation in scRNA-seq effectively. All gene expression profiles which are not influenced by dropouts, would be altered by ScImpute^15^, MAGIC^17^ and SAVER^16^, which might potentially introducing additional biases in the data and perhaps obliterate important biological variance based on probabilistic mixture model. By contrast, VIPER pertains a sparse non-generative regression model to impute zero values in gene expression levels in the cells of interest. Similarly, DrImpute interestedly anticipated dropouts from technical /true zeros counts more precisely in addition to identifying similar cells by clustering their corresponding expression values. Additionally, more recent development of neural network imputation models for instance SEDIM^22^, GE-Impute^23^, AutoImpute^18^, DCA^24^, scScope^25^, scvis^26^, DeepImpute^27^, GSCI^28^ and PBLR^29^, which exploits dropout layers adopting loss functions besides clustering to resolve dataset patterns. The above models preferentially improved the clustering performance of scRNA-seq data rather than considering aspects such as classification, DEA and visualization; instead, they mostly focused on overcoming the sparsity problem and precisely use the bottleneck feature for downstream analysis. The latent features of scRNA-seq data might be distorted and noisy if hidden code is not constrained during the feature learning process, which is not helpful for downstream analysis.

**Figure 1:**
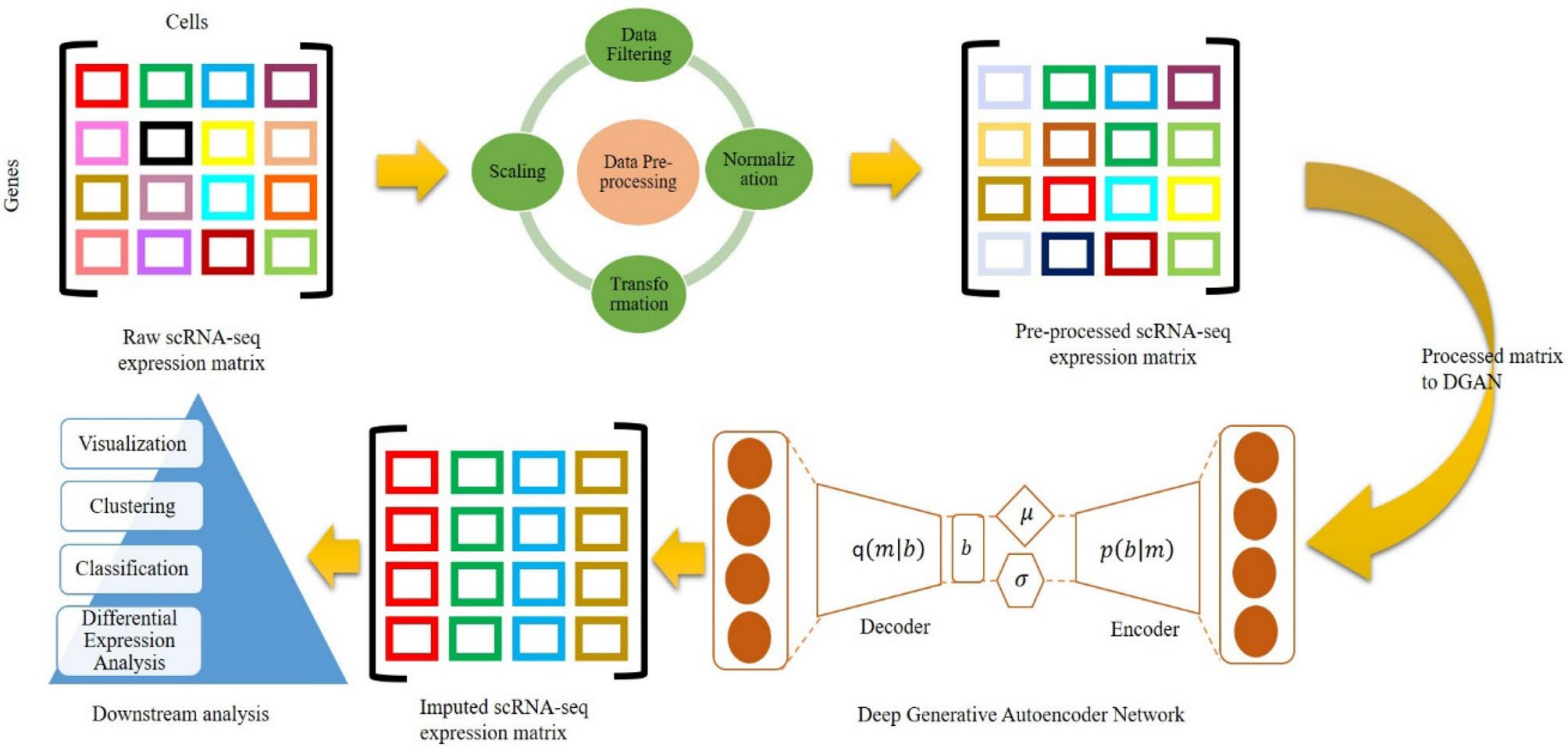
Schematic of deep generative autoencoder network (DGAN) downstream functional analysis pipeline for scRNA-seq data: The real input matrix ‘m’ is filtered for bad genes, normalize them according to library size and pruned by log transformed and scaling. The processed matrix is then fed into the DGAN model, which learns gene expression data depiction and reconstructs the imputed matrix. Finally, these imputed matrix facilitate extensive downstream analysis.

In this study, we have proposed a stacked neural network inspired framework labelled as Deep Generative Autoencoder Network (DGAN) (Fig. 1). In essence DGAN is a revamped Variational Autoencoder (VAE)^30^ based imputation model intended principally for noisy scRNA-seq experimental data. DGAN mechanistically attempts to catalogue the real scRNA-seq data into expediently compressed subsets, thereby evolving a learning model of the intrinsic data distribution masked in the real data. Utilizing a sparse gene expression imputation matrix, here we demonstrate DGAN’s relative performance paralleled with contemporary imputation methods such as DeepImpute^27^, DCA^24^, GSCI^28^, and PBLR^29^. While imputation of technical zero counts significantly improved DGAN’s estimation efficiency, nevertheless as a means to assess the latent ability of DGAN we have chosen both real and imputed data as inputs. From our relative analysis DGAN exhibited significant improvements in all of the downstream functional analyses including visualization, clustering, classification and differential expression analysis. Additionally, with reference to performance, accuracy and memory usage we observed DGAN performs better.

## Materials and methods

### ScRNA-seq data selection and pre-processing

Dual archives i.e. National Centre for Biotechnology Information Sequence Read Archive (NCBI-SRA) database the largest publicly available repository of high throughput sequencing data https://www.ncbi.nlm.nih.gov/sra and 10 × Genomics datasets https://www.10xgenomics.com/resources/datasets were accessed manually on following customized inclusion and exclusion criteria.

In accordance with the search criteria five published and publicly accessible scRNA-seq datasets (Table 1) were selected. The complete datasets were accepted and retained for subsequent pre-processing and representational downstream functional analyses. For ease of comprehension, all of the aforementioned datasets were assigned unique dataset tags, henceforth denoted as Karen, Zeisel and Basile, PBMC, HEK293T-NIH3T3.

**Table 1:** List of datasets selected for representational downstream functional analyses.

Dataset tags	Platform	Organism	No. of genes	No. of cells	References
Karen	10 × Genomics	*H. sapiens*	21,193	1024	^31^
Zeisel	STRT-Seq	*M. musculus*	14,499	3005	^32^
Basile	10 × Genomics	*H. sapiens*	18,967	2366	^33^
PBMC	NovaSeq	*H. sapiens*	15,223	1150	^34^
HEK293T-NIH3T3	10× Genomics	*H. sapiens M. musculus*	32,545	1007	^35^

### Download and pre-processing of the scRNA-seq datasets

Prior to the pre-processing step the Fast-q dump tool https://rnnh.github.io/bioinfo-notebook/docs/fastq-dump.html#fastq-dump was implemented for downloading sequencing reads from NCBI-SRA database and 10 × Genomics datasets. The sequence reads were downloaded and stored as FASTQ files. As the initial phase of pre-processing, the quality of all raw sequencing reads was inspected adopting FASTQC quality control tool for high throughput sequence data^36^. Subsequently, so as to process the reads along with reference annotations, the essential gene annotations were downloaded for both human and mouse genomes besides transcriptome sequences from Ensemble FTP server i.e. Genome assembly GRCh38.p6 release 97 for human and GRCm38.p6 release 97 for mouse. Hitherto, refinement and interlacing of expression data in a uniform format has been executed besides rescaling for data ranges exceeding 100 log transformation (base 2). Normalization of the expression matrices data was conducted by dividing each read count in each cell by its total count, and the median read count across the cells were added. In each set of expression matrix, the top genes with the highest variance were retained for imputation and subsequent downstream functional analysis. Besides reduction of dimensionality of the expression datasets, random attributes were too removed in the pre-processing phase.

### Deep generative autoencoder network (DGAN)

Characteristically autoencoders belong to self-supervised neural networks that learn to model identity, i.e. by training itself to learn one segment of input from a different segment of the same input eventually both input and output are anticipated to be identical^37^.

However, a key difficulty with autoencoders pertaining to generate the output data is that the bottleneck vector converts their inputs where their encoded vectors lie, consequently, the input may not be continuous nor may permit easy interpolation of the output^38^.

While prior investigations have established collaborative filtering^39,40^ as a probable solution in the amelioration of the problem to a certain extent, however here we have considered an adapted alternative i.e. to implement variational autoencoder based imputation to capture data distribution of noisy gene expression data and consequently, reconstruct a comprehensive denoised version of the same implementing DGAN.

### Mathematical model development

The architecture of DGAN entails a simplified expression matrix m = {x^1^, x^2^,…, x^n^} as input, where cells are denoted by rows likewise genes / transcripts are represented by columns. DGAN comprises three components namely a probabilistic encoder (E), a compressed bottleneck vector (b) and a probabilistic decoder (D). The input matrix m is thereby transformed in to a gaussian distribution comprising of mean ($\mu$) and covariance ($\sigma$) by means of the probabilistic encoder (E). Moreover, the bottleneck vector (b) is sampled from the gaussian distribution which is in essence a compressed version of the input expression matrix. The probabilistic encoder (E) is denoted in the form of a mathematically expression below.

$$ {\text{E }} = p_{\Psi } \left( m \right) $$

$$ {\text{b }} = \mu \left( m \right) + \sigma \left( m \right) $$

where ψ is the number of weights and biases. µ and $\sigma$ are the mean and covariance respectively.

It may be noted that in the above mathematical expression, discerning sampling from a gaussian distribution is unattainable due to the non-existence of obligatory parameters in the mathematical expression. This is achieved by implementing reparameterization of the above expression which permits restructuring of the mathematical model path to the extent that the random variables are moved outer of the derivative, otherwise inherent randomness of these variables can lead to much larger errors. The reparametrized mathematically expression of bottleneck vector (b) is given below:

$$ b = \mu + \sigma *\epsilon ; \; sample\; \epsilon \; from\; N\left( {0,1} \right) $$

Further, the probabilistic decoder (D) attempts to obtain the denoised version of the input matrix m from the compressed bottleneck vector (b), the probabilistic decoder (D) is denoted in the form of a mathematically expression below.

$${\mathrm{D }= q}_{\delta }\left(b\right)$$

where $\delta$ is the number of weights and biases. Subsequently the probabilistic decoder (D) deploys the denoised version of the input matrix m.

### Assessment of DGAN’s ability in predicting the outcome

Subsequent to model development and optimization were executed by minimizing the error function. DGAN’s error function is composed of two components, namely (1) generative loss and (2) bottleneck loss. While generative loss equates input and output of the model, conversely bottleneck loss which is denoted by Kullback–Leibler divergence (KL-D)^41^ compares the gaussian distribution and the bottleneck vector, i.e. bottle neck loss specifies the similarities between the two distributions.

$${e}_{m}\left(\Psi ,\delta \right)=-{KL}_{D}\left[{p}_{\Psi }\left(m\right)\| {q}_{\delta }\left(b\right)\right]+E{p}_{\Psi \left(m\right)}\left[log({q}_{\delta }\left(b\right))\right]$$

where $${KL}_{D}\left[{p}_{\Psi }\left(m\right)\| {q}_{\delta }\left(b\right)\right]= {E}_{b\sim p}\left[log({p}_{\Psi }\left(m\right))-log({q}_{\delta }(b))\right]$$

### DGAN implementation and hyperparameters

DGAN was executed adopting Python3 with TensorFlow^42^ as backend. To perform imputation, we have implemented Adam^43^ optimizer with hyperparameters including learning cost (0.001), batch size (100), number of epoch (50), besides encoder’s dimension, hidden dimension, vector dimension, and decoder’s dimension. For the model development, the value of hyperparameters were set as per the obligation of real dataset. Masking was introduced to manage missing, invalid/unwanted entries in the datasets. The hardware specification of the system configured is as follows: Intel(R) 8-core processor, 16-GB RAM, 500-GB hard drive and × 64 base system.

### Relative downstream functional analysis of scRNA-seq data

#### Data visualization

Data visualization was executed by deploying the Violin plot function from the ggplot2 package in R^44^. Violin plot integrates both box plots and histogram together to illustrate the distribution and median of data. Additionally, Violin plot represents data of models in terms of log of coefficient of variation. Violin plot illustrates parameters including interquartile range, median and whiskers that demonstrate larger interquartile ranges.

#### Cell clustering

For Cell clustering analysis Seurat^45^ package which was implemented in R. Seurat is capable of predicting both spatial cell clustering and localization. For this study, Seurat was adopted for rendering the spatial location of the entire transcriptome besides detecting rare subpopulations within the expression matrix including the numerical count of genes, cells and genes expressed in each cell. Three evaluation metrics including Adjusted rand index (ARI)^46^, Fowlkes mallows index (FMI)^47^, Silhouette coefficient (SC)^48^ were considered. ARI is a modified version of the Rand index defined by $ARI=\left(RI-E\left[RI\right]\right)/\left(1-E\left[RI\right]\right)$, where E denotes expected and the R and index (RI) measures similarities between the two data clusters and ARI is an adjustment for chance groupings. Likewise, FMI pertains to clustering performance metric for evaluating the cluster’s similarities obtained and calculated based on false negatives (FN), false positives (FP) and true positives (TP).

FMI has been explained as follows: $\sqrt{\frac{TP}{TP+FP}}.\frac{TP}{TP+FN}$

To conclude, the silhouette score was adopted to estimate the mean silhouette coefficient with a range between -1 and 1 and the mean of intra-cluster (x) and nearest-cluster distance (y) as $\left(y-x\right)/max\left(x, y\right)$ was calculated.

Non-linear dimension reduction methods, such as Principal Component Analysis (PCA)^49^, t-Distributed Stochastic Neighbor embedding (t-SNE)^50^ and Uniform Manifold Approximation and Projection (UMAP)^51^ predominantly intend at grouping similar cells in a low-dimensional space. Subsequently, Cluster identification was executed in the following stages (1) normalizing and scaling of data (2) linear dimension reduction by PCA (3) calculating the dimensionality of datasets and (4) clustering of cell subpopulations applying Louvain algorithm optimization.

#### Classification

We implemented multi-class classifiers to classify scRNA-seq data into different categories^52^. Both linear and non-linear models were considered for classification including Logistic Regression (LR)^53^, Support Vector Machine (SVM)^54^, Random Forest (RF)^55^, Naive Bayes (NB)^56^, K-Nearest Neighbor (KNN)^57^, Decision Tree (DT)^58^ and Gradient Boosting (GB)^59^. Subsequent division of the input gene expression matrix data into training and testing data all the aforementioned classification algorithms were implemented. As part of each analysis scenario, dataset was divided into 70% training and 30% testing in classification model. Training data were used to determine the most effective composition of hyperparameters by the grid-search manner and to estimate their performance, while independent predictors were based on testing data.

While plenty of metrics such as accuracy, recall, confusion matrix, precision, F1-score and ROC curve prevail two most frequently implemented metrics namely accuracy and AUC-ROC curve were implemented. While accuracy measures how often the classifier correctly predicts, i.e. the proportion of true results among the total number of cases examined. Consequently, both accuracy and Area Under the Curve- Receiver Operating Characteristic (AUC-ROC) curve was considered for the model’s classification performance evaluation metrics^60^. Based upon the confusion matrix^61^, we calculated true negative (TN), TP, FN, and FP and then computed accuracy was calculated as follows:

$$ {\text{Accuracy}} = \frac{{Number\;of\;correct\;predictions}}{{Total\;number\;of\;predictions}} $$

where number of correct predictions was calculated as [TP + TN] and total number of predictions was calculated as [FN + FP + TN + TP].

AUC-ROC curve was implemented to visualize the multi-class classification model performance. ROC curve was designed by plotting True Positive Rate (TPR) on the y-axis and False Positive Rate (FPR) on the x-axis.

#### Differential gene expression analysis

Differential gene expression (DGE) analysis is one of the most detailed methods to identify dysregulations of gene/transcript under different subpopulations or cell types^62^. DGE analysis was adopted utilizing a negative binomial generalized linear model DESeq2^63^. Read count data in the form of matrix were programmed as input for DESeq2 package. The raw counts were normalized implementing size factors and the estimated gene-wise dispersions were contracted to generate more accurate estimates of Log2 Fold Change (Log2FC) for the model adopting Wald test.

As a result, a matrix of differentially expression genes was generated encompassing Log2FC, basemean, adjusted values (padj) and pvalue. For visualization of the topmost differentially expressed genes (DEG) identified by DESeq2 in R, we have implemented a scatter plot^64^ to exhibit the correlation between numeric variables, a whisker plot^65^ to display the summary of the dataset and a heatmap^66^ to graphically represent the selected (DEG) in an assortment of colours.

## Results

### Enhancement in visualization of imputed data

**Figure 2:**
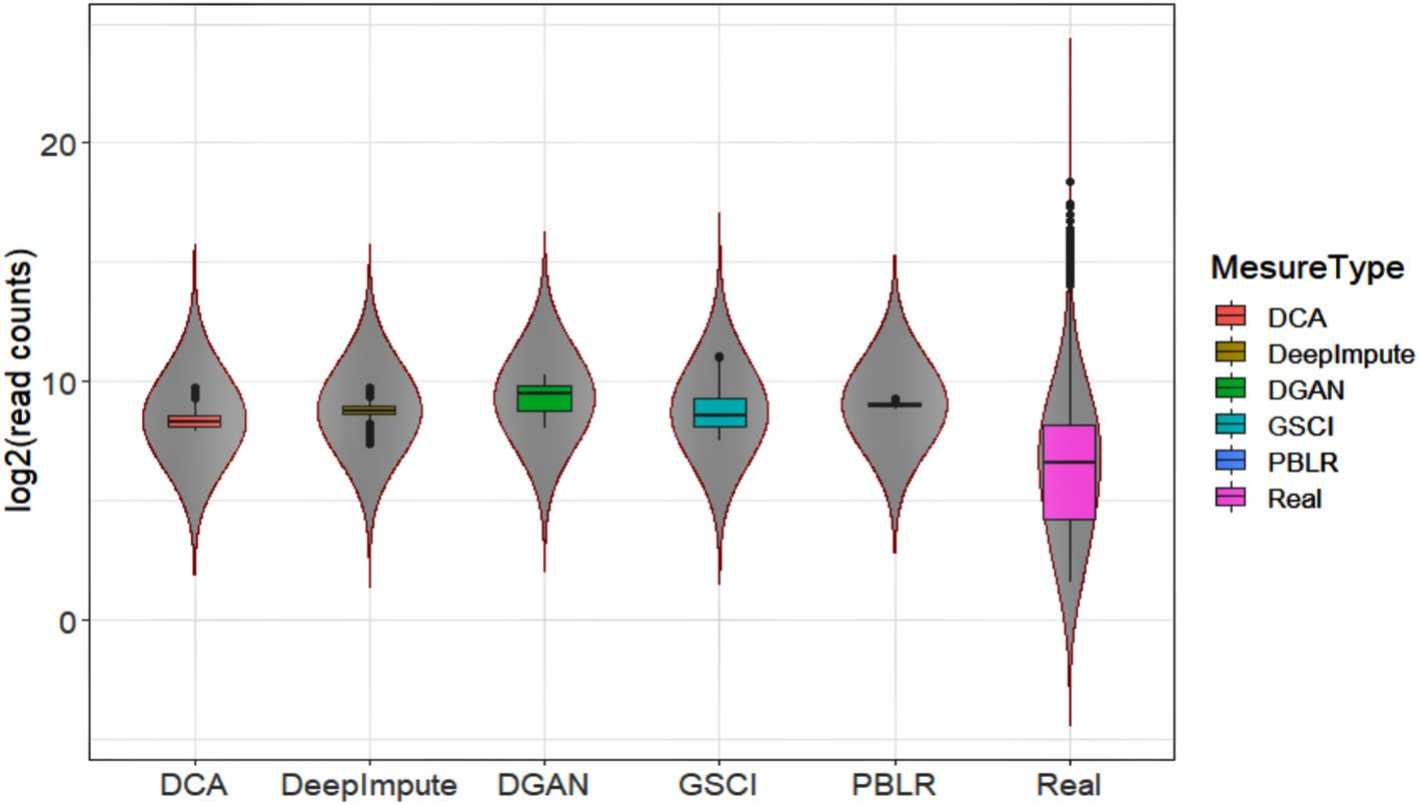
Violin plot depicting real and imputed data of Basile dataset attained from implementing all paralleled models in terms of log of coefficient variation computed for individual genes across the cells. The interquartile range is represented by the box, in addition the median is represented by horizontal line and whiskers demonstrate larger interquartile ranges.

In order for an imputation to be equitable, the gene expression should be reduced within subpopulations. We scrutinized cellular gene expression variance from a randomly selected Basile dataset. The gene expression levels are displayed via violin plot^44^ that include a marker for the median and as in a normal box plot, the box indicates the interquartile range, which allow users to compare how each gene is expressed across a wide range of diverse cellular subtypes and determine its kernel probability density easily. A reasonable DGAN imputation done on real dataset to recover the expressive transcriptome dynamics in biological single cells. It was found that DGAN and GSCI^28^, the variance in gene expression within subpopulations has almost been stabilized for Basile^33^ performs better than all imputation methods except DeepImpute^27^, DCA^24^ and PBLR^29^ in Fig. 2. It depicts the summary statistics and the peak density of each variable of Basile for all comparative models. It found that DGAN gives a reasonable improvement in coefficient of variation. More outlier has been seen in DeepImpute^27^, DCA^24^ and PBLR^29^ likened with DGAN model which clearly indicates our DGAN model removed the noise data present in input scRNA-seq data. Similarly, the gene expression levels of the other datasets with DGAN model are included separately in Fig. S1.

### Denoising improved in clustering analysis

**Figure 3:**
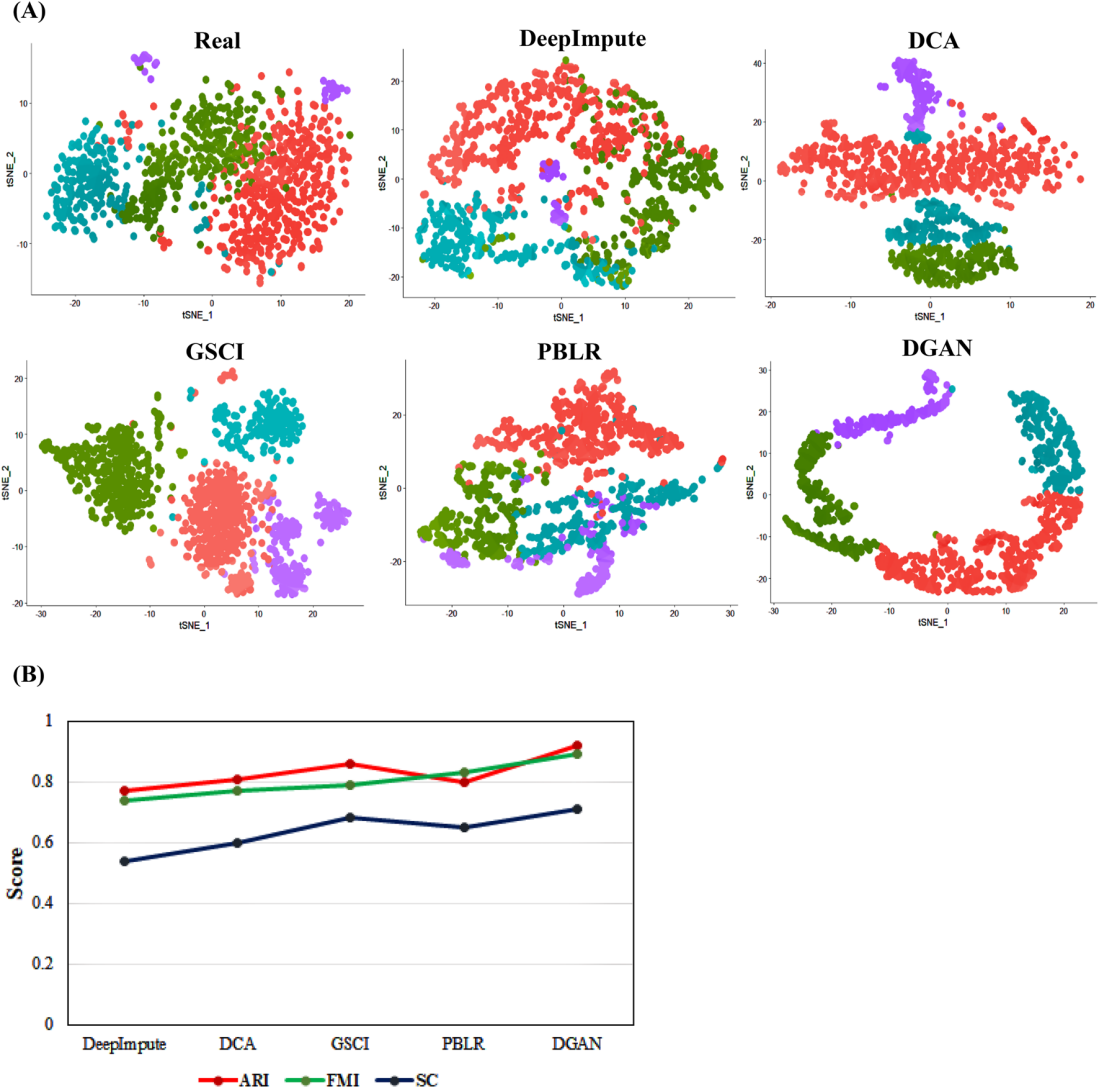
Clustering analysis; (**A**) Representative visualization of clusters determined by t-SNE 2D visualization method for pre-imputed (Real) Karen scRNA-seq dataset. Imputed matrix via DeepImpute, DCA, GraphSCI, PBLR and DGAN. The cells colours are assigned according to their cell groups. (**B**) ARI, FMI, and SC signify clustering evaluation performance of scRNA-seq data of DeepImpute, DCA, GraphSCI PBLR and DGAN respectively.

Dropouts and missing values are a key concern in large scRNA-seq datasets including those attained from whole tissues. Besides resulting in inappropriate expression levels^67^ dropouts and missing values also cause hassle in clustering of the data as most clustering algorithms are vulnerable. To investigate this problem, the impact of denoising on clustering were examined. While clustering of real data is inherently difficult due to noisiness of data therefore, we executed clustering evaluation metrices on imputed data to define the robustness and effectiveness of paralleled methods. A systematic comparison of denoising attained by DGAN as compared to DeepImpute^27^, DCA^24^, GSCI^28^ and PBLR^29^ is enclosed in Table S1. To obtain gene expression projections using t-SNE^50^ as observed it gives better visualization than PCA^49^ and UMAP^51^, we compared the Karen^31^ dataset having 21,193 genes and 1024 cells with different selected denoised models, and clustered the cells using the Louvain algorithm as shown in Fig. 3A. Through visualization, the clusters obtained from DeepImpute^27^, DCA^24^and PBLR^29^ methods were mixed with each cluster where DGAN separated the four clusters clearly. Although GSCI^28^cope to split numerous cell clusters, its dispersion of data in Fig. 3A is highly distorted. Moreover, the precision of clustering assignments has been calculated using numerous evaluation metrics counting the Adjusted Rand Index (ARI)^46^, the Fowlkes-Mallow Index (FMI)^47^, and Silhouette Score (SC)^48^ to exam t-SNE^50^ clusters (Fig. 3B). On the divergent, DeepImpute^24,27^ and DCA^24^ decrease, rather than improving the clustering outcome. As shown in Fig. 3B, DGAN attained 0.92, 0.89 and 0.71 for ARI, FMI and SC values. These results are better than the results achieved by DeepImpute^27^, DCA^24^, GSCI^28^ and PBLR^28^. Based on the evaluation metrics, DGAN achieves virtually perfect scores for ARI, FMI, and SC which is significantly higher than the other models. Although both DeepImpute^27^ and PBLR^28^ have a small amount of cells varied together, DGAN clearly separates four types of cells. The real data can’t parse out the cells. In both clustering and metrics methods DGAN outperforms than other.

### Retrieval of mRNA signals in scRNA-seq real data

Another important factor to appraise the clustering techniques is their capability to recuperate mRNA gestures in real scRNA-seq data set to show improvisation of clustering with DGAN. Therefore, we have chosen other two different real scRNA-seq datasets named Zeisel^32^ and HEK293T/NIH3T3^35^ with different number of cell counts and sequencing protocols used for our method for clustering structures. We tested the visualization performance of DGAN along with three non-linear dimension reduction techniques, including PCA^49^, t-SNE^50^ and UMAP^51^ together in Seurat package^45^. For this analysis, we compared the clustering results of both real and DGAN dataset in Fig. S2(A) to (C). While identifying the dimensionality of the dataset, extract the significant principal components (PCs) with higher standard deviation which help to find which cells exhibit similar expression patterns for clustering and resolution. With all datasets, a parameter resolution in Seurat setting between 0.6 and 1.2 produces good outcomes. However, increasing the resolution increases the number of clusters. Cells are color-coded according to their PCA scores for each respective PC during cell visualization. The Fig. S2(A), (B), (C) of Karen^31^ DGAN, Zeisel^32^ DGAN and HEK293T/NIH3T3^35^ DGAN shows data representation clearly, which consists of cells of the same type grouped together and of the different types separated from each other, along with we discovered that it has a good number of markers which could be used for further downstream analysis. On the other hand, in Fig. S2(A), (B), (C) of real data we can observe that most cells are overcrowded, low quality, and overlapping. Also, the overall result was undesirable since the cells of different types did not compactly cluster together and therefore could not provide better visualization in the dataset. In overall, the DGAN disentangles many clusters, leading in the most enhanced clustering metrics compared with the scenario without DGAN. According to the experimental results of each datasets in clustering analysis, we found that for Karen^31^ our model has better outcome than other dataset.

### Improvisation of cell classification in scRNA-seq datasets

In order to prove our method's principle and investigate its properties, we tested the classification on imputed scRNA-seq data generated using different imputation models such as DCA^24^, GSCI, PBLR^28,29^ and our DGAN. DeepImpute^27^ was excluded from the comparison, due to insufficient processing time and memory.

To examine DGAN's classification ability, we compare it with seven methods that are predominant in machine learning: Logistic Regression (LR)^53^, Support Vector Machine (SVM)^54^, Random Forest (RF)^55^, Naive Bayes (NB)^56^, K-Nearest Neighbor (KNN)^57^, Decision Tree (DT)^58^ and Gradient Boosting (GB)^59^. We tested these methods on Zeisel^32^ dataset. In this 3005 cells and 14,499 genes were profiled from the STRT-Seq platform. The scalability and robustness of DGAN were demonstrated on the large-scale scRNA-seq dataset by applying all four imputation models. As part of each analysis scenario, our dataset was divided into 70% training and 30% testing in classification model. Based on training data, optimal hyperparameters have been identified and their performance has been estimated, while independent predictors were based on testing data. To optimize the classification model performance evaluation metrics^60^ should be calculated. There are plenty of metrics such as accuracy, recall, confusion matrix, precision, F1-score and ROC curve but in this analysis we have applied most frequently used accuracy and AUC-ROC curve.

**Figure 4:**
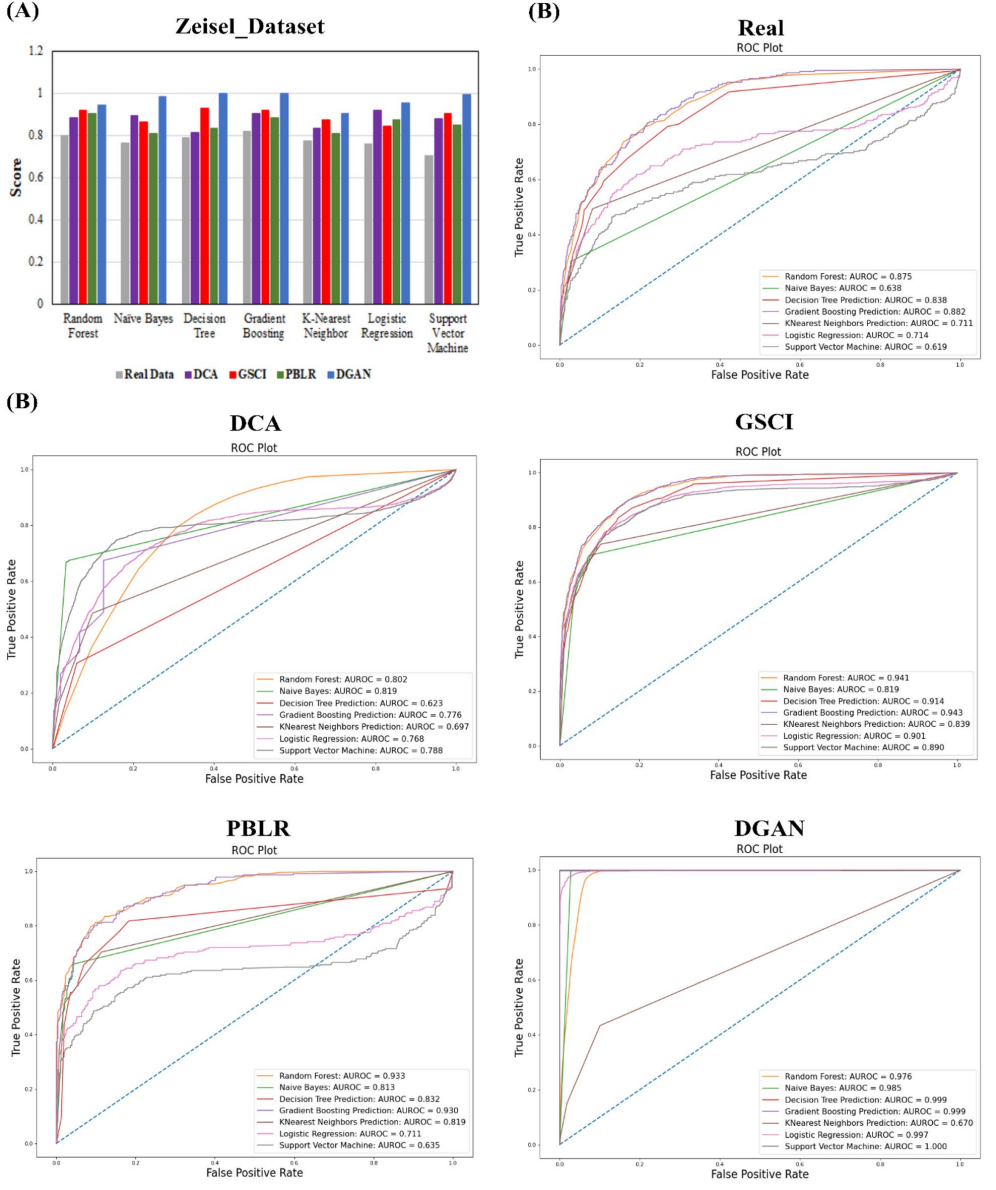
(**A**) The performance graph is of Zeisel dataset where individual colour bars represent different real data and imputed data from DCA, GSCI, PBLR and DGAN models. (**B**) AUC-ROC measurements of various classification algorithms. AUC-ROC measurements of imputation built on different models and individual line colours representative of different algorithms.

Figure 4A and Table S2 show the accuracy of each method. Accuracy measures how often our classifier correctly predicts, it is the proportion of true results among the total number of cases examined. Model with accuracy rate of 99% considered a good model and vice versa. Overall, DGAN has an accuracy of 0.90 to 1.0 across all combinations. With the highest accuracy, DGAN outperforms all other methods. DGAN's average accuracy is 0.96 compared to 0.77, 0.87, 0.89, and 0.85 for real, DCA, GSCI and PBLR respectively. Furthermore, the performance of DGAN is consistent, in contrast to existing models, which are not consistently accurate, particularly when the training dataset is considerably larger than the testing dataset. AUC-ROC curve of all mentioned methods with Zeisel^32^ dataset are shown in Fig. 4B. It defined how well the probabilities from positive classes are separated from negative classes for a range of different cut-off points. Given that the decision threshold under AUC default 0.5 suggest that the classifier is not able to distinguish between positive and negative classes whereas higher the threshold upto 1, better the performance of the model. As a result, it is evident that AUC-ROC score is higher for DGAN (Fig. 4B) compared to other models. As we can see, AUC-ROC for DGAN is the better model to distinguish the cells by covering the larger area whereas other models are struggle to distinguish, the blue line shows the threshold means the classifier predicts either constant or random class for whole data points. In Fig. 4B for PBLR model, the SVM^53,54^ and LR^53^ values fall below the blue line, similar behaviour observed for DCA model.

### DGAN enriched classification over scRNA-seq real data

To assess the performance of DGAN over different classification algorithms, we experimented on two more scRNA-seq datasets, PBMC^34^ and Karen^31^ and compared their real and DGAN datasets through mentioned seven classification algorithms. Figure S3 shows the accuracy score of classification algorithms executed on above declared real datasets and its DGAN data. By comparing the real over DGAN datasets (Fig. S3) (Table S3), it clearly seen that classification algorithms gives better accuracy results for DGAN data with range of 0.9 to 0.92. In addition, Random Forest (RF)^55^ outperforms other algorithms by having the highest accuracy for DGAN dataset. The average accuracy of DGAN dataset covering all classification methods is close to 0.92, whereas for real dataset is 0.79. Moreover, an ensemble voting of tools on PBMC^34^ DGAN data presented a slightly better accuracy, which provide a new thought to correctly classify single cells with high similarity.

To investigate more on accuracies, we performed the ROC analysis to evaluate whether the classification capabilities of tools are diverse for different cell types. AUC-ROC curve of all methods for two real and DGAN dataset are shown in Fig. S4(A) and S4(B) for PBMC^34^ and Karen^31^dataset. As a result, it is evident that AUC-ROC score is higher for PBMC^34^ and Karen^31^ DGAN data compared to their real datasets. Furthermore, Random Forest (RF)^55^ topped algorithm for DGAN data among its competitors having on average decision threshold of 0.9. Among three used datasets, Zeisel^32^ gives good metric under ROC curve. As an inference, based on evaluation metrics the classification underwent a greater improvement when using DGAN model rather than imputation model.

### Imputation and convalescent gene expression of scRNA-seq data

**Figure 5:**
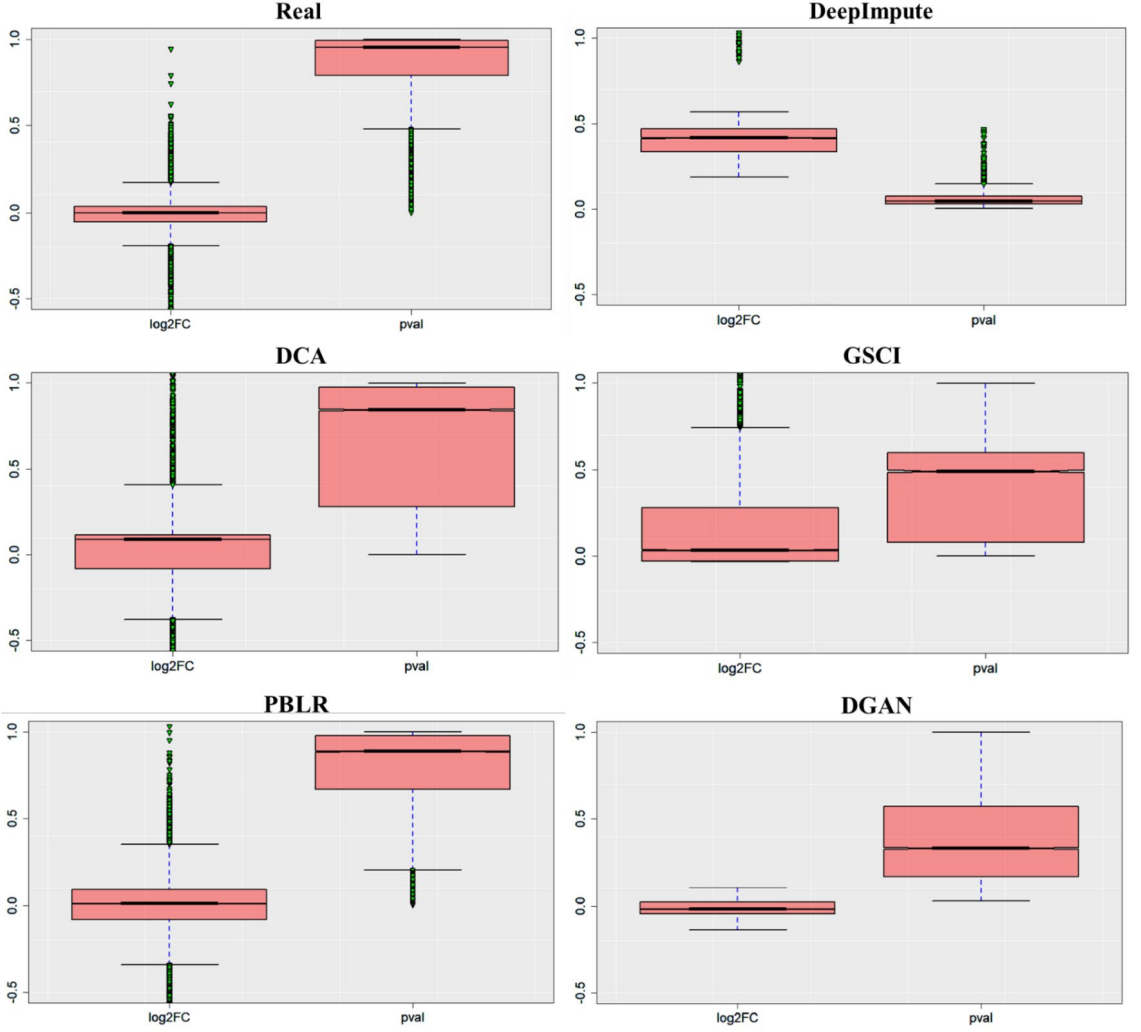
Performance of DGAN on large-scale dataset, whisker plot of gene expression for log2FC and pval by differential expression analysis using PBMC data with different models.

DGAN can’t only impute in scRNA-seq data effectively, but also enhance differential expression analysis (DEA). To assess whether DGAN can identify DEGs more accurately after imputation of scRNA-seq dataset compared to DeepImpute^27^, DCA^24^, GSCI^28^, and PBLR^29^. These models were applied on healthy donor dataset PBMC^34^ extracted from NovaSeq including 15,223 genes and 1150 cells and performed DEA on the real versus imputed data correspondingly using DESeq2^63^ package. DESeq2 uses an empirical Bayesian approach to integrate dispersion and fold change estimates, and use the Wald test to determine DEGs based on the assumed log-normal distribution for each gene. There are plenty of visualization method for DESeq2, out of those we selected whisker plot^65^ as it gives more information about the outliers. The plot (Fig. 5 and Table S4) depicts the Log2FC, pvalue as usual logarithmic value of the gene covariance across cell subtypes using PBMC^34^ data across all the imputation model including our DGAN. The whisker plot measures the probability of the data being well distributed by dividing it into three quartiles minimum, maximum, median where first quartile, and three quartile are identified. In Fig. 5 some distribution for models such as DeepImpute^27^, DCA^24^, GSCI^28^ and PBLR^29^ are widely spread around the medium values in addition there are more data points beyond the limit of minimum and maximum values identified as triangle with green colour is treated as outlier unlikely in DGAN, data is closely distributed and most of the data points fall within the limits.

### Data-driven differential expression analysis with DGAN

For determining whether DEGs identification after imputation is more accurate, we used more two scRNA-seq datasets such as Basile^33^ and Zeisel^32^ with DGAN outcomes and compared the available statistical techniques for differential expression analysis (DEA) to produce biologically precise results. The visualization of these datasets through DESeq2 package achieved by regularized logarithm transformation tools, namely scatter plot, whisker plot and graphical heatmap in Fig. S5(A) to (C). We compared the performance of above methods on real and DGAN dataset which help to find the topmost differential expression marker genes. An effective multivariate visualization technique, scatter plot matrix, which plots read count distributions across all samples and genes. We plotted Log2FC, pvalue and padj for presenting discrete observations.

As compare to real data, in DGAN data most genes should fall in the 3D space within default threshold as we expect only a small proportion of them to show differential expression between samples are shown for PBMC^34^, Basile^33^ and Zeisel^32^ DGAN in Fig. S5(A) to (C). The scatter^65^ plot for DGAN data display a higher correlation among the three numerical variable. A set of data variable is distributed over the scatter plot for real but appears to cluster for DGAN. Moreover, we applied whisker plot in both real (Basile and Zeisel) and with its DGAN data respectively and observation is like seeing less outliers for DGAN data compared to real data of selected dataset in Fig. S5(B) and (C). Coming to the last DESeq2 visualization tool, to determine subcategories within an experiment, it is often helpful to plot the DEGs as a heatmap^66^ where colors are used for graphical representation, which allows us to visualize features and samples simultaneously. Using DESeq2, we examined the differential expression of genes after removing low expression genes with threshold of fold change ≥ 0.02 between cells, alongside with a *p* value ≤ 0.05 after padj correction.

From Fig. S5(A) to (C), the DEGs in each group were visualized along with all parameters using heatmap with real and DGAN data. Then, we likened differences in real gene expression upon DGAN dataset, it is perceived more common values or higher activity with brighter colour is more with DGAN data correspond to real data, where darker colour indicates less expressive genes. The platter of heatmap related to DGAN data of PBMC^34^ and Basile^33^ has darker shade than DGAN data of Zeisel^32^. All together, these results show that DGAN allows for an advance in downstream DESeq2 functional analysis based on real and DGAN data.

## Discussion

Besides alleviating computational complexity, imputation efficacy considerably influences downstream functional analysis especially when dropout levels are particularly high as is the case with droplet-based technologies^68^. The larger the proportion of missing values, the more demanding the imputation task. However, scRNA-seq technology opens up many possibilities for single-cell resolution analysis using deep learning algorithms^69^. Inspired by the recent success of artificial neural networks, we proposed an imputation model based Variational Autoencoder, the DGAN model. Our model focused on estimating patterns of gene expression levels in individual cells by projecting expression profiles into a low-dimensional bottleneck vector, and has advantages in downstream functional analyses, including visualization of gene expression landscapes, clustering of cell types, cell classification and differential expression analysis. Unlike existing Autoencoders and statistical impute models such as DeepImpute^27^, DCA^24^, GraphSCI^28^, PBLR^29^and SAVER^16^, scImpute^15^ and scMTD^21^ that was developed for data imputation and with a drawback of not applying Gaussian distribution in the bottleneck vector^70^, DGAN provides a complete analysis pipeline from pre-processing to dimension reduction to imputation and downstream analysis. The existing imputed methods show limited number of functional downstream analysis. To our finest knowledge, this is the first attempt to inherently distribute scRNA-seq data by applying gaussian distribution along with reparameterization technique in bottleneck vector of neural network framework for imputation and downstream functional analysis using implementing a state-of-the-art deep learning approach. Additionally, DGAN is essentially "buoyant" i.e. model trained with a subset of input data, nevertheless still could make out decent predictions, which is in a way beneficial, as it can further reduce the overall execution time. As an alternative, DGAN assumes only those dropout entries that are most likely to occur across cells based on a mixture model. However, because of the non-linear relationships and including structures, the scRNA-seq datasets cannot be learned by models such as scMTD^21^ and SAVER^16^. A given data distribution assumption is normally used for above statistical models and scImpute, in case of non-conformance, the completion effect will be degraded.

An important aspect of DGAN is that it is scalable, which makes it more realistic and feasible for large-scale variational inference datasets. DGAN is a statistically generative model while other comparable models can be considered to be compressor and decompressor models. In addition to single point modeling, DGAN has several additional parameters to tune to better fit our latent space (probability distribution). DGAN represents latent variables with detangled factors due to their isotropic Gaussian priors, which allow each dimension to grow as far away from each other as possible. As well as regularizing the effect of the prior, DGAN also adds a regularization coefficient. Further, as paralleled with other imputation models DGAN offers a comprehensive analysis pipeline starting off with pre-processing, dimensionality reduction, imputation and follow-up downstream functional analysis including visualization, clustering, classification, and differential expression analysis. Based on these results, it is clear that DGAN is an extremely effectual and accurate method for imputation, which is likely to remain applicable for the foreseeable future due to scRNA-seq data volume growth. Five real scRNA-seq datasets were imputed implementing the DGAN model and the performance of the model was evaluated with various downstream functional analyses as compared with other contemporary models^71^. To test the reliability of our model, we randomly nominated three datasets for each functional downstream analysis and while comparing with other imputation models, we arbitrarily selected only one dataset from above three. Also, selected only those visualization method for DGAN and its other comparative models from each downstream analysis which gives better conception about dataset such as t-SNE^50^ for clustering, AUC-ROC for classification^52^ and whisker plot^65^ for differential expression analysis. As a result, DGAN achieves the better imputation visualization with Basile^33^ data over other persisting models in Fig. 2 and Fig. S1. Real data also poses alternate challenge as clustering would be problematic due to noisiness and the absence of ground truth. Hence, we evaluated competitive methods using clustering evaluation metrics to describe their effectiveness and robustness, as well as visualized the results to make them more comprehensible. Indeed, GE-Impute^23^ and SEDIM^22^ are the most recently published imputation models for scRNA-seq data analysis. While GE-Impute is based on graph embedding neural network model, SEDIM proposed an automatic design of deep neural networks architecture. Both the models perform imputation, yet they are diversified from DGAN over the algorithms adopted, still we have attempted to compare the clustering efficiency evaluation metrics. Since existing scRNA-seq imputation methods focus on identifying cells or genes that are similar, they rarely consider gene–gene relationships and correlations into account, making it impossible to retain biological variation across cells or genes. Clustering downstream analysis is ubiquitous amongst DGAN, GE-Impute and SEDIM, as with evaluation performed based on ARI and UMAP. GE-Impute and DGAN performed almost analogously with ARI of 0.93^23^ and 0.92 respectively, whereas SEDIM performed relatively not as much of with 0.73^22^. In Addition, UMAP clusters seems to be clearly separable in DGAN compared with above models. Based on Fig. 3A,B we found that t-SNE^50^showed better outcomes and the performance of DGAN is consistently improved with a variety of clustering approaches. Moreover, using a single set of hyperparameters, DGAN imputed data achieves the highest accuracies and AUC-ROC score of classification model, amidst existing model, GSCI^28^ surpassed and its score lie to DGAN where Random Forest^55^ outperform as compared to different machine learning methods in Fig. 4A,B. Alternative noteworthy aspect of our verdicts is the biological relevance of topmost gene expression levels between experimental datasets. To find the topmost gene expression levels with different datasets, we performed differential expression analysis using Bayesian approach for each datasets (Fig. 5). In addition to that, the results were visualized in three different methods, namely scatter plot^64^, whisker plot^65^and heatmap^66^ graphical representation of colours. The DGAN data come out as centred, garner, less skewness in scatter plot, with negligible outliers in whisker plot and topmost marker genes in heatmap. As gene expression levels increase in scRNA-seq data, DGAN has been perceived to improve a higher number of noisy events than other imputation models and superior enhancement in downstream functional analysis.

## Conclusion

An ever-increasing amount of dropout cells and technical noise, all of which characterize high-throughput scRNA-seq data, pose important challenges in downstream functional analysis^70,72^. Dealing with very sparse expression matrices compromises the accuracy and scalability of the analysis and severely obstruct our ability to extract the vast amount of usable information from single-cell data. However, scRNA-seq technology opens up several possibilities for single-cell resolution analysis using deep learning algorithms^73^. Inspired by the recent success of artificial neural networks, we have proposed an imputation model based Variational Autoencoder, dubbed DGAN model. Our model focused on estimating patterns of gene expression levels in individual cells by projecting expression profiles into a low-dimensional bottleneck vector, and has rewards in downstream functional analyses, including visualization of gene expression landscapes, clustering of cell types, cell classification and DEA. As far as we know, this work is one of a kind and probably the first to inherently distribute scRNA-seq data in an artificial neural network framework for imputation and downstream functional analysis implementing a state-of-the-art deep learning approach. More importantly, extensive comparative investigations were performed on diverse scRNA-seq datasets to demonstrate the influence of our method as compared to contemporary state-of-the-art methods. While our focus was set on single-cell analysis, it is our modest opinion that with minor amendments DGAN could be implemented for a wide range of high-throughput data applications.

Based on our experimental outcomes, DGAN is a proof-of-concept demonstration that bias could be eliminated adopting a standard matrix recovery method combined with downstream functional analysis besides signifying scRNA-seq pipeline can be integrated seamlessly.

## Supplementary Information

Supplementary Information 1.

Supplementary Information 2.

## Data Availability

All the raw ScRNA-seq datasets have been retrieved from NCBI Sequence read archive (SRA) and the 10 × Genomics webpage. Two of them (PBMC^31^ and HEK293T-NIH3T3) were taken from 10 × Genomics that is PBMC^34^ https://www.10xgenomics.com/resources/datasets/10-k-pbm-cs-from-a-healthy-donor-v-3-chemistry-3-standard-3-0-0 and HEK293T-NIH3T3^35^ https://www.10xgenomics.com/resources/datasets/1-k-1-1-mixture-of-human-hek-293-t-and-mouse-nih-3-t-3-cells-3-v-3-1-3-1-standard-6-0-0. Other three datasets were downloaded from NCBI SRA such as SRP247631^31^ https://www.ncbi.nlm.nih.gov/Traces/study/?acc=PRJNA605373&o=acc_s%3Aa, SRP045452^32^ https://www.ncbi.nlm.nih.gov/Traces/study/?acc=PRJNA258094&o=acc_s%3Aa and SRP260978^33^ https://www.ncbi.nlm.nih.gov/Traces/study/?acc=PRJNA631512&o=acc_s%3Aa.
