# Supplementary material for: Improved downstream functional analysis of single-cell RNA-sequence data using DGAN
Source: Sci Rep. 2023 Jan 28;13:1618. doi: 10.1038/s41598-023-28952-y (PMC9884242; doi:10.1038/s41598-023-28952-y)
Supplement: Supplementary file 1 — Supplementary Information 1. [file 41598_2023_28952_MOESM1_ESM.docx]

**Supplementary Information**

**Supplementary Figure and Tables with legend**

**Figure S1:**

**
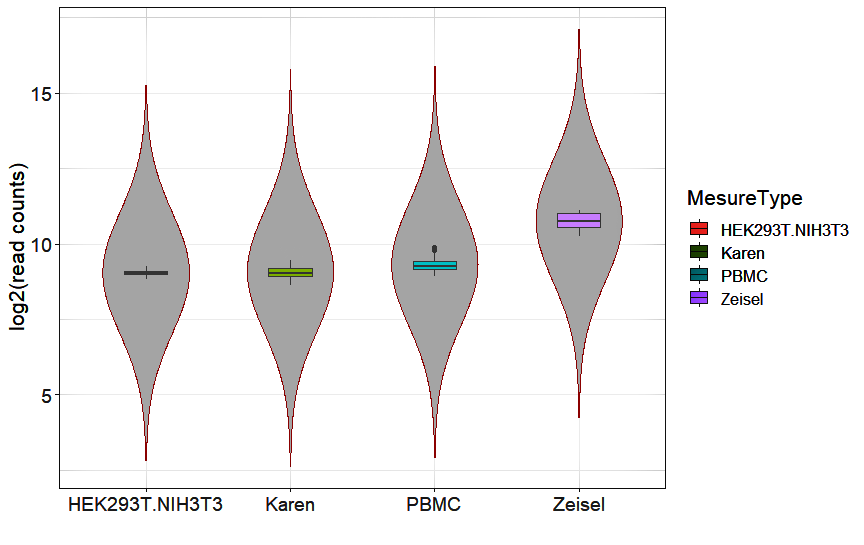
**

**Figure S1:** The performances of gene expression levels of imputed datasets.


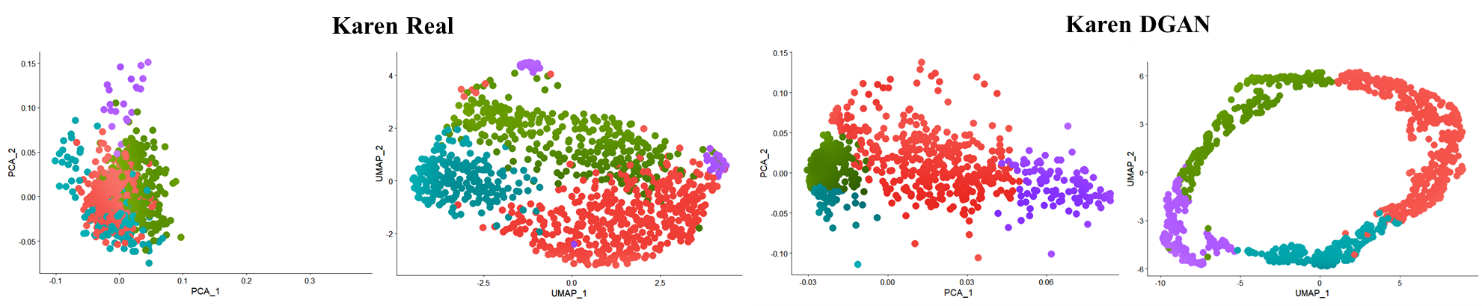


**Figure S2:**

**(A) ()**

**(B)**


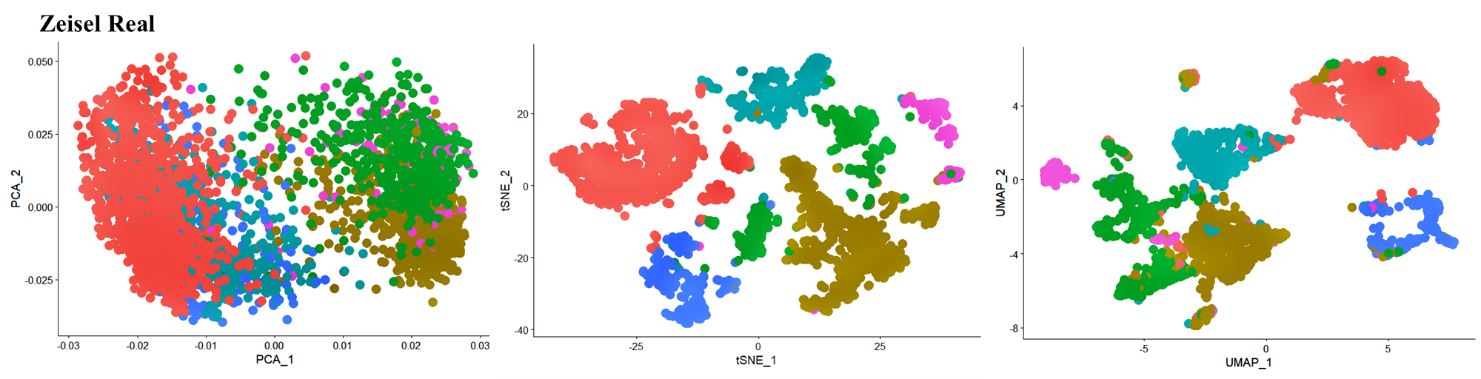


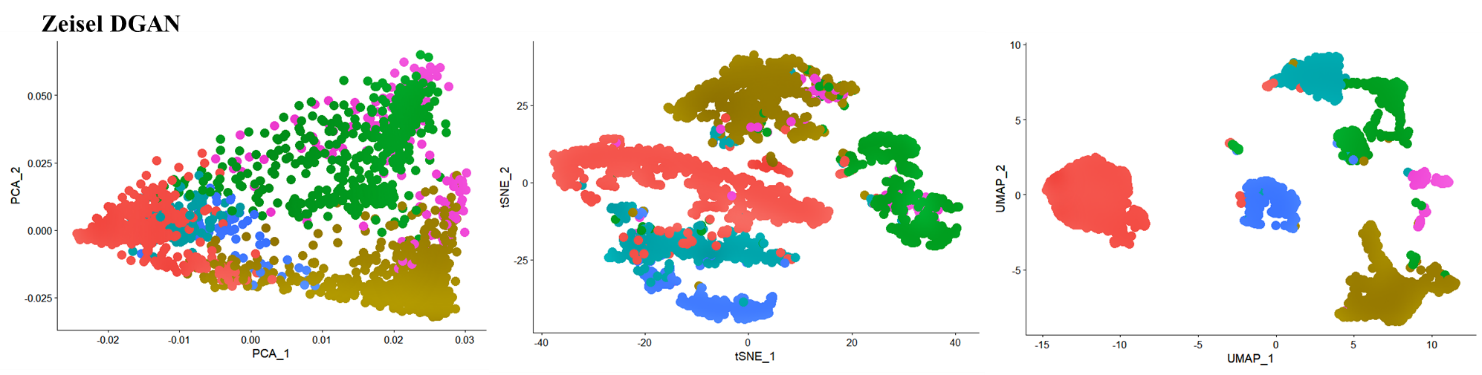


**(C)**


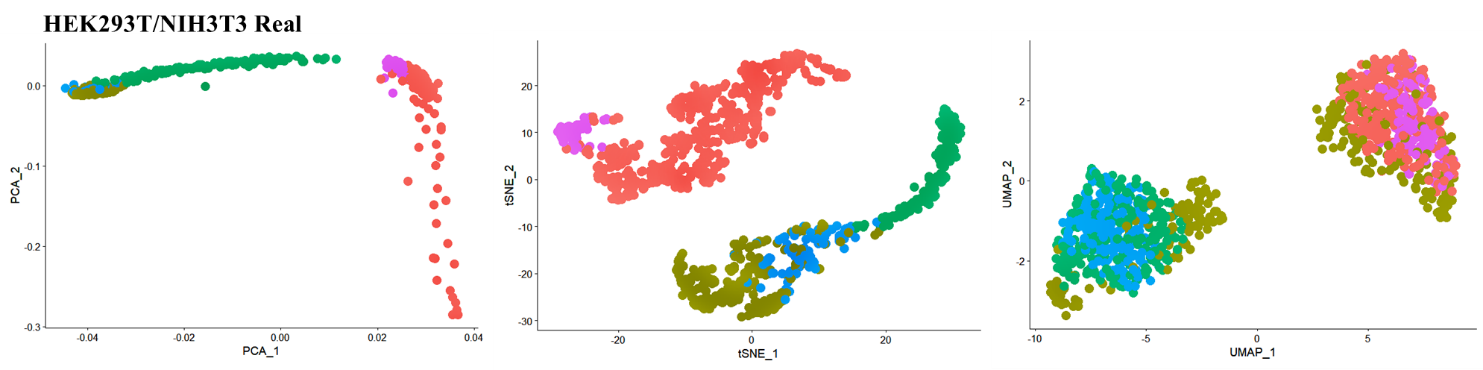


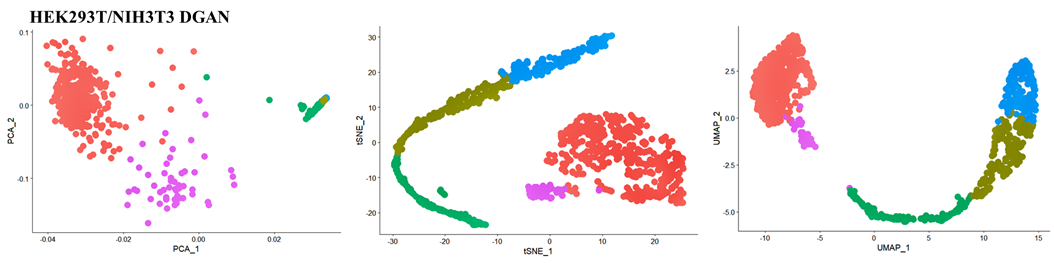


**Figure S2:** Clustering performance visualization pertaining to real and imputed datasets utilizing 2D visualization methods such as PCA, t-SNE and UMAP; (A) Cluster visualization for Karen_ real and Karen_DGAN (B) Zeisel_real and Zeisel_DGAN (C) HEK293T/NIH3T3_real and HEK293T/NIH3T3_DGAN.


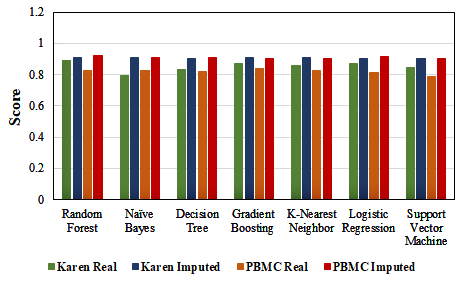


**Real & DGAN**

**Figure S3:**

**Figure S3:** Exhibit accuracy of real and imputed datasets using seven different classification methods on PBMC and Karen datasets.

**Figure S4:**

**(A)**


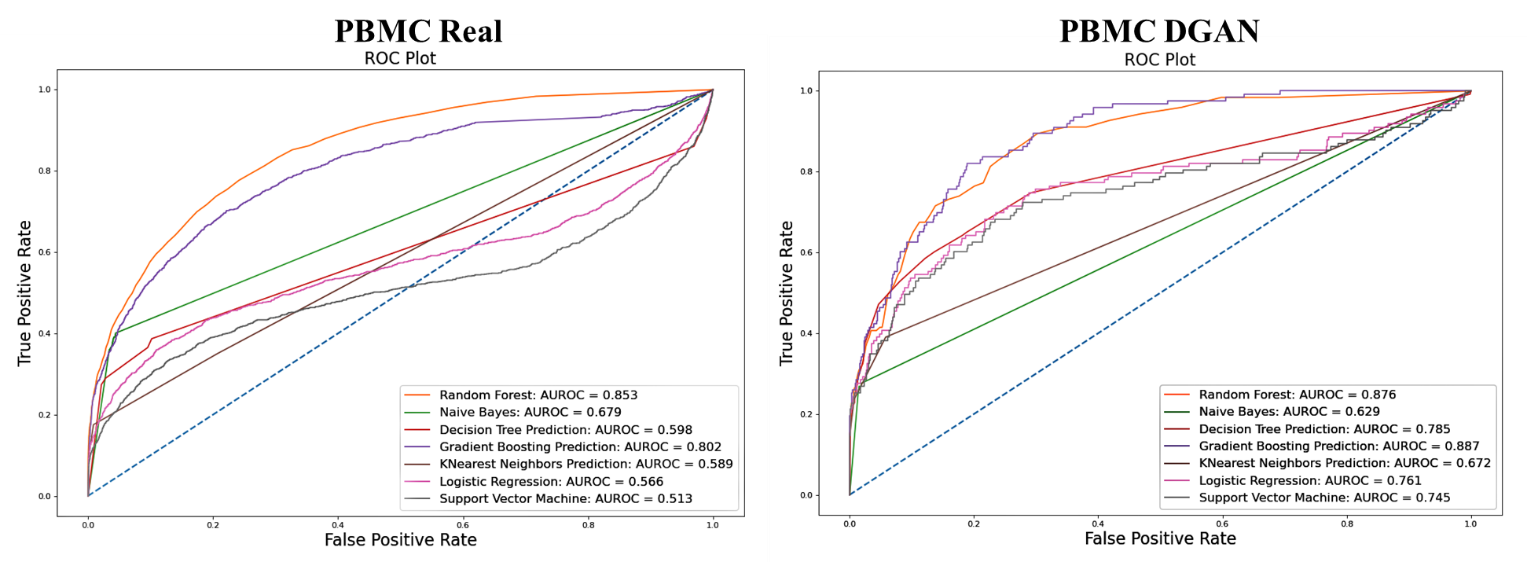


**(B)**


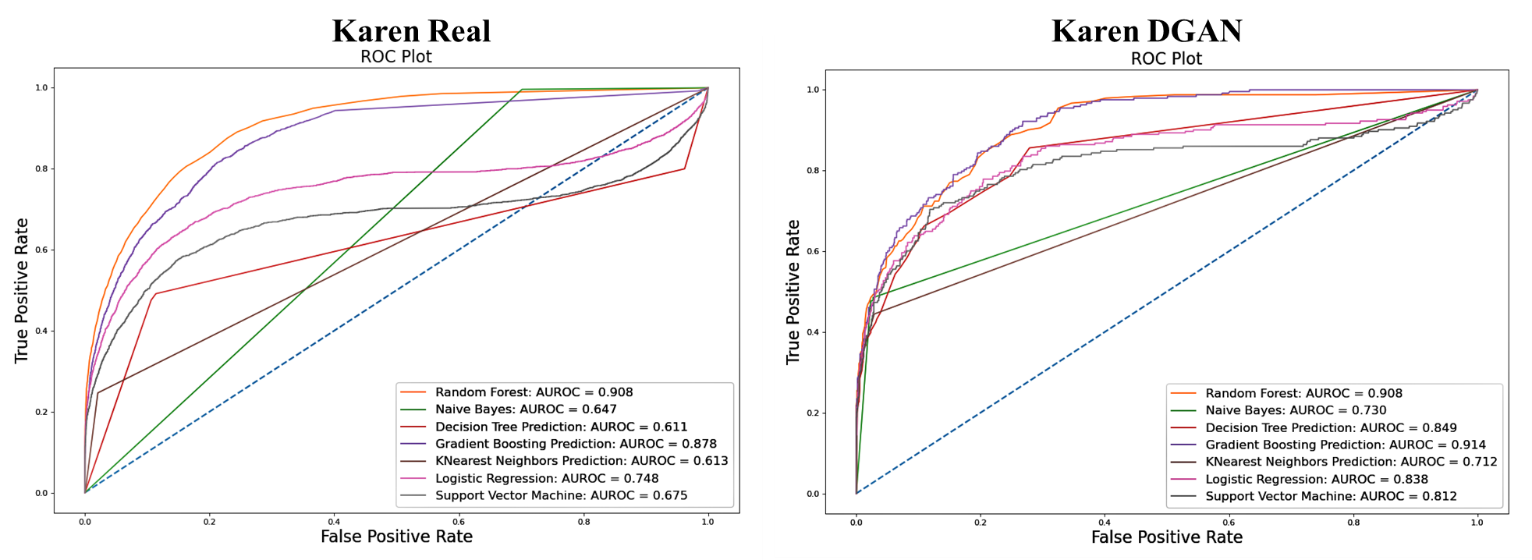


**Figure S4:** (A) & (B) Depict the AUC-ROC plot for real and imputed datasets. The curve depicts the comparison between different classification methods measured by ROC score.


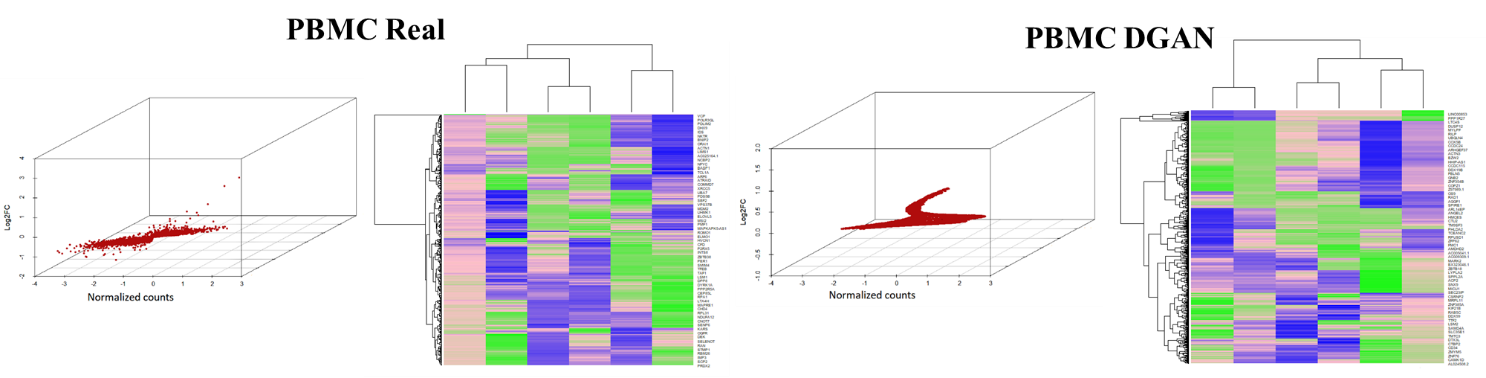


**(A)**

**Figure S5:**


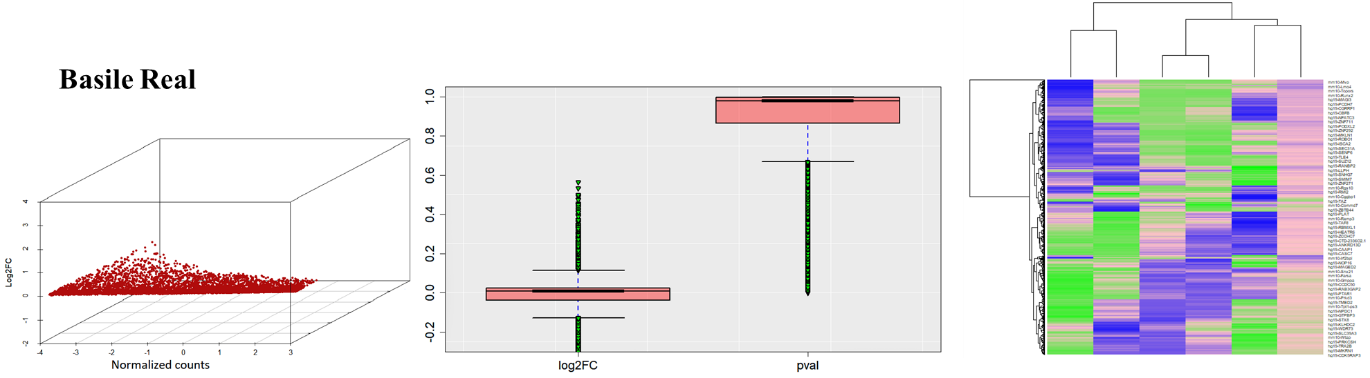


**(B)**


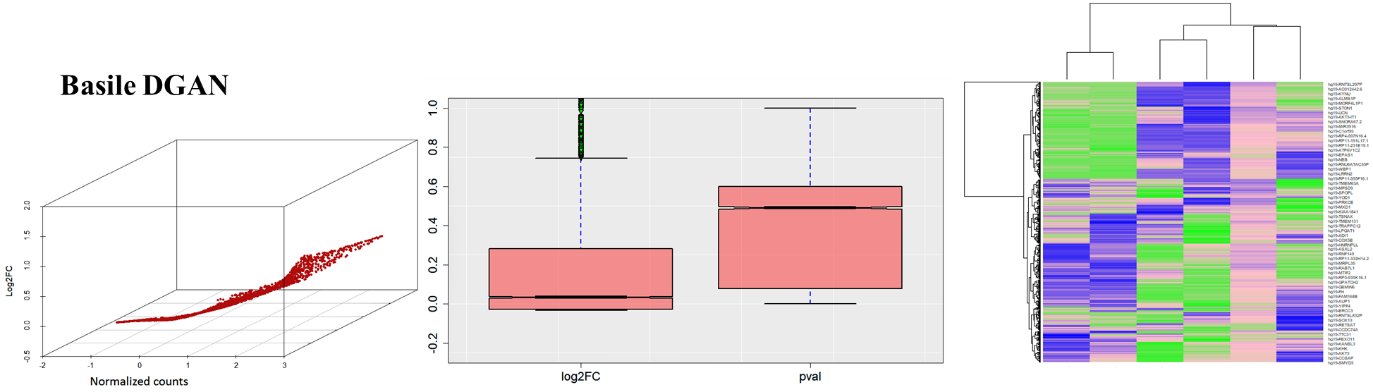


**
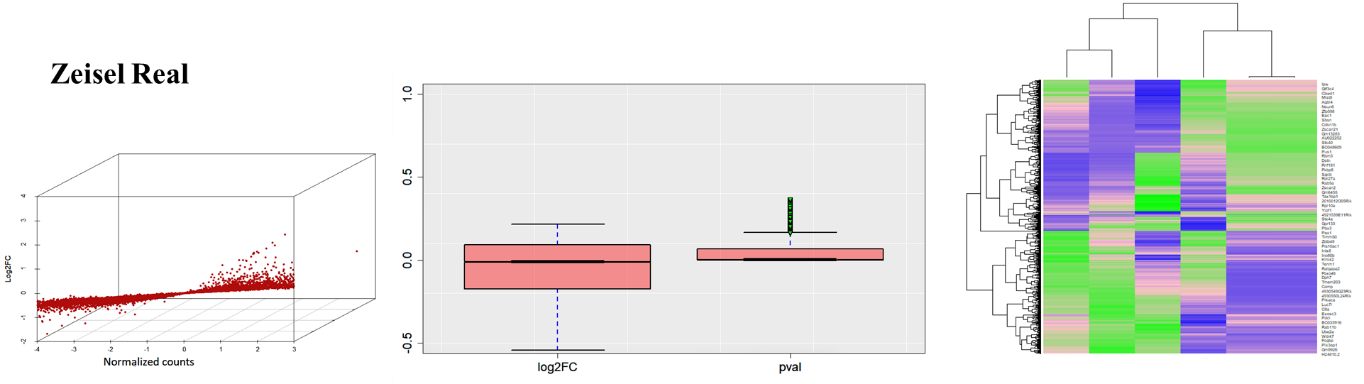
**

**(C)**

**
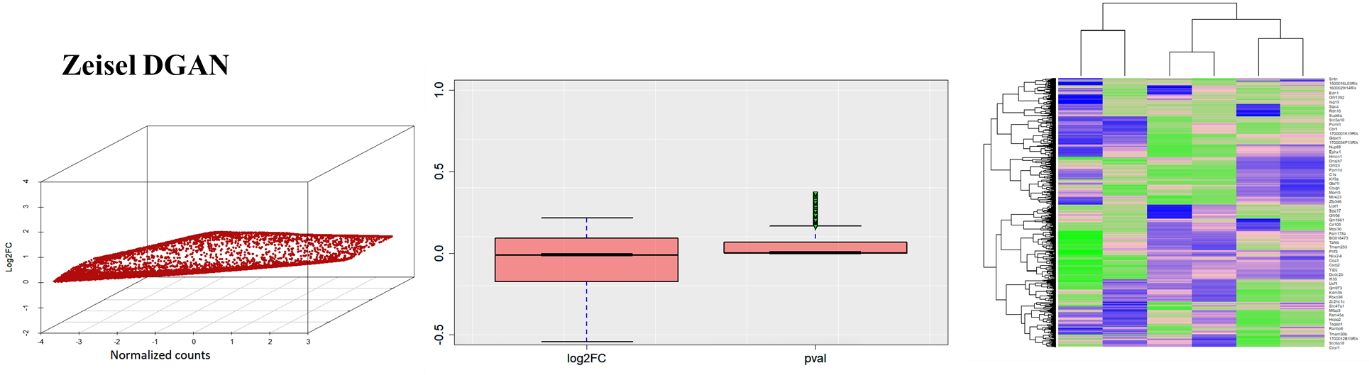
**

**Figure S5:** Representation of DEA of three scRNA-seq datasets using R (v4.1.2) language based functions with R-Studio (v2021.09.2+382) IDE available at (*RStudio Team (2022). RStudio: Integrated Development for Environment R. RStudio, PBC, Boston, MA URL*[*http://www.rstudio.com/*](http://www.rstudio.com/)*.*); (A) Scatter plot and graphical representation heat map top expressed genes where individual values are represented as higher activity colours for PBMC real dataset. (B) & (C) depict the scatter plot, whiskers and heatmap compared between real and imputed data of Basile and Zeisel datasets.

**Table S1: The Results of Karen dataset related to Figure 3B**

| **Karen_Evaluation metrics** | | | | | |
| --- | --- | --- | --- | --- | --- |
| **E_metrics** | **DeepImpute** | **DCA** | **GSCI** | **PBLR** | **DGAN** |
| **ARI** | 0.77 | 0.81 | 0.86 | 0.8 | **0.92** |
| **FMI** | 0.74 | 0.77 | 0.79 | 0.83 | 0.89 |
| **SC** | 0.54 | 0.6 | 0.68 | 0.65 | 0.71 |

**Table S2: Accuracy of Zeisel dataset related to Figure 4A**

| **Accuracy** | | | | | |
| --- | --- | --- | --- | --- | --- |
| **Score** | **Real Data** | **DCA** | **GSCI** | **PBLR** | **DGAN** |
| **Random Forest** | 0.79647 | 0.88294 | 0.91785 | 0.90161 | 0.94305 |
| **Naïve Bayes** | 0.76375 | 0.89227 | 0.86254 | 0.80643 | 0.98181 |
| **Decision Tree** | 0.78586 | 0.81427 | 0.92973 | 0.83357 | 0.99983 |
| **Gradient Boosting** | 0.81654 | 0.90412 | 0.91635 | 0.88463 | 0.99695 |
| **K-Nearest Neighbor** | 0.77276 | 0.83568 | 0.87214 | 0.80926 | 0.90204 |
| **Logistic Regression** | 0.75962 | 0.91693 | 0.84346 | 0.87361 | 0.95458 |
| **Support Vector Machine** | 0.70519 | 0.87587 | 0.90543 | 0.84862 | 0.99092 |
| **Avg accuracy** | 0.7714 | 0.8745 | 0.8925 | 0.8511 | 0.9670 |

**Table S3: Accuracy of Karen and PBMC datasets related to Figure S3**

| **Real vs Imputed** | | | | |
| --- | --- | --- | --- | --- |
| **Score** | **Karen Real** | **Karen Imputed** | **PBMC Real** | **PBMC Imputed** |
| **Random Forest** | 0.89161 | 0.91142 | 0.82894 | 0.92237 |
| **Naïve Bayes** | 0.79643 | 0.9109 | 0.82558 | 0.9071175 |
| **Decision Tree** | 0.83357 | 0.90303 | 0.81881 | 0.911713 |
| **Gradient Boosting** | 0.87463 | 0.9088 | 0.84122 | 0.904 |
| **K-Nearest Neighbor** | 0.8596 | 0.90828 | 0.82505 | 0.901713 |
| **Logistic Regression** | 0.87361 | 0.90566 | 0.81632 | 0.914664 |
| **Support Vector Machine** | 0.84862 | 0.90618 | 0.79085 | 0.9066119 |
